# Calcium plus vitamin D_3_ supplementation facilitated Fat loss in overweight and obese college students with very-low calcium consumption: a randomized controlled trial

**DOI:** 10.1186/1475-2891-12-8

**Published:** 2013-01-08

**Authors:** Wei Zhu, Donglian Cai, Ying Wang, Ning Lin, Qingqing Hu, Yang Qi, Shuangshuang Ma, Sidath Amarasekara

**Affiliations:** 1 Department of Nutrition, Shanghai Institute of Health Sciences, Shanghai, 201318, China; 2 Department of Clinical Nutrition, Changhai Hospital, Second Military Medical University, Shanghai, 200433, China; 3 Department of Clinical Nutrition, Chengdu Military General Hospital, Chengdu, 610083, China

**Keywords:** Calcium, Vitamin D_3_, Adiposity, Body weight, Body fat, Visceral fat, Metabolic profiles

## Abstract

**Background:**

Recent evidence suggests that higher calcium and/or vitamin D intake may be associated with lower body weight and better metabolic health. Due to contradictory findings from intervention trials, we investigated the effect of calcium plus vitamin D_3_ (calcium+D) supplementation on anthropometric and metabolic profiles during energy restriction in healthy, overweight and obese adults with very-low calcium consumption.

**Methods:**

Fifty-three subjects were randomly assigned in an open-label, randomized controlled trial to receive either an energy-restricted diet (−500 kcal/d) supplemented with 600 mg elemental calcium and 125 IU vitamin D_3_ or energy restriction alone for 12 weeks. Repeated measurements of variance were performed to evaluate the differences between groups for changes in body weight, BMI, body composition, waist circumference, and blood pressures, as well as in plasma TG, TC, HDL, LDL, glucose and insulin concentrations.

**Results:**

Eighty-one percent of participants completed the trial (85% from the calcium **+** D group; 78% from the control group). A significantly greater decrease in fat mass loss was observed in the calcium **+** D group (−2.8±1.3 vs.-1.8±1.3 kg; *P*=0.02) than in the control group, although there was no significant difference in body weight change (*P*>0.05) between groups. The calcium **+** D group also exhibited greater decrease in visceral fat mass and visceral fat area (*P*<0.05 for both). No significant difference was detected for changes in metabolic variables (*P*>0.05).

**Conclusion:**

Calcium plus vitamin D_3_ supplementation for 12 weeks augmented body fat and visceral fat loss in very-low calcium consumers during energy restriction.

**Trial registration:**

ClinicalTrials.gov (NCT01447433, http://clinicaltrials.gov/).

## Background

Dietary calcium, a non-energy-supplying nutrient, has been identified as playing a pivotal role in the regulation of energy and lipid metabolism [1]. Observational studies have demonstrated calcium intake is inversely associated with body weight [2-4], dyslipidemia [1], type 2 diabetes [5] and hypertension [6]. In the same vein, higher vitamin D intake and elevated level of serum 25(OH)D have been reported to be related to lower adiposity [7,8] and metabolic health [9]. However, results from randomized controlled trials to evaluate the effect of supplemented calcium with or without vitamin D on weight management and metabolic profiles remain controversial [10,11].

Findings from Zemel’s study of the agouti gene in obesity and insulin resistance demonstrated that increased intracellular calcium, resulting from a low calcium diet, resulted in stimulation of lipogenic gene expression, which increased lipogenesis and inhibited lipolysis, thus promoting adiposity [12]. It is suggested, however, that vitamin D deficiency increases appetite and decreases energy consumption by stimulating Agouti Related Protein/ Neuropeptide Y (AgRP/NPY) and suppressing the pro-Opiomelanocortin/ Cocaine- Amphetamine- Regulated Transcription (POMC/CART) pathway [13].

One plausible explanation for the contradictory findings could be the threshold hypothesis, suggested by Zemel et al. and Major et al., that promising outcomes might be predicted under the cut-off value of initial calcium intake (500–600 mg/d) in a calcium supplemented weight-loss program [10]. We initiated the current study in subjects whose calcium consumption was below the cutoff value, with the hypothesis that greater weight reduction and a better metabolic profile would be seen in the calcium+D supplemented group than in the control group, independent of caloric deficit.

## Methods

### Study design

We conducted an open-label, randomized, parallel-group controlled trial from April to December, 2011. Recruitment and screening procedures were launched in the first 6 months, after which a 12-week intervention period was carried on.

### Participants

Participants were recruited through flyers and advertisements posted on college campuses in Pudong and Yangpu Districts in Shanghai, China. A total of 129 volunteers were evaluated for eligibility. Participants of the present study had to be generally healthy (absence of coronary heart disease, hypertension, diabetes; dyslipidemia), overweight or obese (defined by a body mass index (BMI) of 24 kg/m^2^ or more and 28 kg/m^2^ or more, respectively, according to the Chinese standard [14-16]), aged 18 to 25 years, daily calcium intake below 600 mg (assessed with a food frequency questionnaire (FFQ)), stable body weight (body weight change less than 1 kg for 2 months before screening), less than 3 times per week of 20 mins of physical exercise, no use of calcium supplements or any medication that could affect body weight within 30 days of screening, no smoking, absence of pregnancy or lactation, and not participating in any weight loss programs or in any other clinical trial within 6 months of screening. Each participant was fully informed of the risks and benefits associated with the study and signed written informed consent form. All subjects were studied under protocols approved by the Shanghai Changhai Hospital Ethnics Committee (CHEC2011-020). This study was registered on ClinicalTrials.gov. (NCT01447433).

### Randomization and interventions

A total of 53 subjects were randomly assigned with a 1:1 allocation to either calcium+D (n=26) or control group (n=27). Randomization was computer-generated, and a randomization sequence was created using SPSS 13.0 for windows (SPSS Inc., Chicago, USA). Stratified randomization was adopted when allocating 7 male subjects, with 3 allocated to calcium+D and 4 to the control group. The calcium+D group received 600 mg of calcium carbonate plus 125 IU vitamin D_3_ (purchased from the primary agent of Caltrate in Shanghai, China), administered as the dose of one tablet taken daily after breakfast.

In addition, both groups received 500 kcal/d of caloric deficit. Total daily energy expenditure (TDEE) of each subject was calculated using World Health Organization equations for calculation of basal metabolic rate (BMR), and was estimated as 1.3×BMR for obese individuals engaged in mild daily activity [14]. Based on this initial estimate of energy needs, a standard recipe of a balanced diet (protein 10~15% of total energy supply, fat 20~30%, carbohydrates 55~65%) was created to produce a daily caloric deficit of 500 kcal. Each participant received a standard recipe and a portion-size picture booklet of food models of common foods and measuring containers for their self-monitoring of daily food intake. Nutritional supplements were not permitted. Dietary instruction and counseling were provided to subjects in both groups during biweekly follow-ups throughout the program.

### Assessment

A semiquantitative FFQ [15] was used for each participant during screening with 149 items in 17 food categories, including rice, wheat flour, other cereals, potatoes, pork, poultry, fish, eggs, milk, legumes and their products, fresh vegetables, salted vegetables, fresh fruits, vegetable oil, nuts, salt, soy sauce and liquor. An average portion size for each item was specified, and subjects were asked about the frequency of consuming that unit throughout the previous year. Dietary records were analyzed, using the Nutrition Calculator Software (version 2.3, developed by the Chinese Center for Disease Control and Prevention [CDC]). Only those whose initial calcium intake was below 600 mg entered further screening procedures.

Prior to randomization, participants received a physical examination, including body weight, height, body composition, blood pressure and blood chemistry. Those who met the inclusion and exclusion criteria entered the program. All of the anthropometric measurements and blood chemistry tests were repeated at the end of 12-wk intervention, while body composition was obtained at 4-wk intervals. All the assessments were obtained using standard protocols.

Anthropometric variables and blood samples were obtained between 8:00 and 10:00 a.m. after an overnight fast. Subjects were asked to void their bladders before measurement and to wear light clothes. Height was measured barefoot using a stadiometer to the nearest 0.1 cm and weight was obtained to the nearest 0.1 kg using a digital scale. BMI was calculated as weight (kg)/ height (m)^2^. Waist circumference were measured using plastic tape to the nearest 0.1 cm. Systolic and diastolic blood pressures were measured in the left arm at heart level with subjects seated for a minimum of 5 minutes using mercurial sphygmomanometer. The measurement was repeated once so as to take the mean value. Bioelectric impedance analysis (BIA, ZEUS 9.9, JAWON) was used to determine body fat mass, fat percentage, and lean mass, as well as visceral fat mass and visceral fat area (VFA). Blood samples were collected and sent to the clinical laboratory of Changhai Hospital within 30 min. Plasma glucose was measured enzymatically. Plasma insulin was assayed using an Electrochemiluminescence Immunoassay Kit (Roche Diagnostics GmbH, Manneheim, Germany). Total cholesterol and triglyceride (TC and TG) and high- and low-density lipoprotein (HDL and LDL) concentrations were measured using an automated enzymatic analyzer (Automatic Analyzer 7080, Hitachi, Tokyo, Japan).

Participants were required to submit 3-day food records at baseline and after 12 weeks. As most of the subjects took their meals at the students’ canteen, where the amount of each dish of food was approximately fixed, we asked them to record the amount of staple food consumed and the portion of the leftover of each dish containing animal or plant foods. The dietitian made a detailed list of each dish of food in advance. Subjects were also asked to record their consumption of fruits, snacks, beverages, etc. Each diet record was carefully reviewed and analyzed by a registered dietitian. Dietary records were analyzed using the Nutrition Calculator Software for energy, protein, fat, carbohydrate and calcium intakes. During the whole period, subjects were required to maintain the same calcium intake as assessed by the initial FFQ. Compliance for total energy and dietary calcium intake were monitored throughout the program while guidance was given to subjects that had trouble following our dietary instructions. Calcium+D tablets were issued at baseline, exchanged for a new package at wk 4 and wk 8, and returned for counting for compliance, which was 95.8% in the calcium+D group, at the conclusion of the intervention.

### Statistical analysis

Statistical analysis was performed by using SPSS 13.0 for windows (SPSS Inc., Chicago, USA). Independent t-test between means and chi-square test for frequencies were used to assess differences between groups in baseline characteristics of subjects. Repeated measures of variance were performed to evaluate the significance of the differences between groups for changes on outcome variables. All data were presented as mean ± standard deviation. *P*<0.05 was considered significant.

## Results

**Figure 1:**
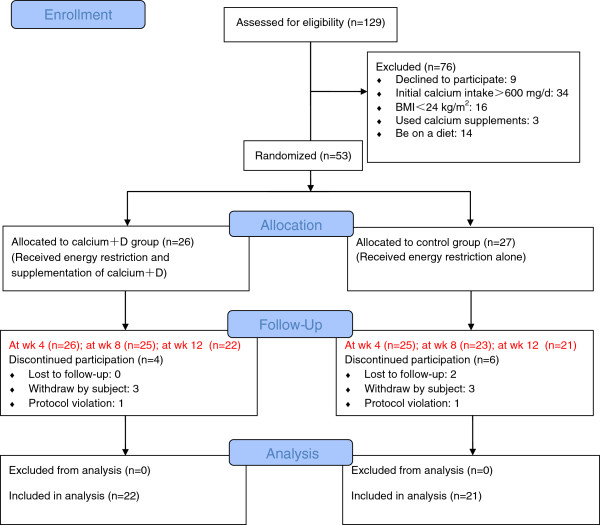
Study flow diagram.

The sample size in both groups was adequate in the present study. We needed a total of 42 participants to detect a 1.5 kg difference in 12 weeks in weight change between groups with 90% power at the 0.05 level. We increased the sample size to 26 per group to allow for dropouts with 20% attrition. A total of 43 subjects completed the trial. Ten dropped out after enrollment: 2 lost contact (both from the control group); 6 withdrew for personal reasons (3 from each group; one dropped out of college, one left for another city for internship, and four lost interest) ; and 2 were excluded for poor compliance (one from the control group that refused to follow our dietary instructions, the other from the calcium+D group for less than 90% adherence by tablet counts at week 8). No adverse event was reported throughout the study. All of the 43 subjects were included in final analysis (Figure 1). 

Baseline characteristics of subjects are shown in Table 1. No significant difference was observed in baseline characteristics between groups. Table 2 indicated anthropometric and metabolic variables at baseline and after 12 weeks, as well as changes in these variables between measurement periods. As expected, both groups achieved significant reduction in anthropometric variables during the 12-wk program in the context of energy restriction. Significant time×treatment effects were observed for changes in body fat mass, fat percentage, visceral fat mass and VFA (*P*<0.05 for all), in which the difference between groups remained significant even after adjustment for age, gender, initial calcium intake and baseline body weight. A significantly greater decrease in fat mass loss was observed in the calcium **+** D group (−2.8±1.3 vs.-1.8±1.3 kg; *P*=0.02), which was 55.6% higher than in the control group, although there was no significant difference in body weight change (*P*>0.05) between groups. Subjects in the calcium **+** D group also exhibited greater decrease in visceral fat mass and VFA (*P*=0.01 and *P*=0.02, respectively). No significant difference was detected for changes between groups in lean mass, waist circumference, or blood pressure, or in plasma TG, TC, HDL, LDL, glucose and insulin concentrations (*P*>0.05 for all). Table 3 showed body composition changes of subjects between baseline and wk 4, wk 8, and wk 12 on the basis of the intention-to-treat (ITT) analysis. As illustrated in Table 3, the calcium + D group witnessed significantly greater reduction in fat mass, fat percentage, visceral fat mass and VFA at wk 8 and wk 12 (*P*<0.05 for all), compared with the control group. 

**Table 1:** Baseline characteristics of subjects

	**Calcium+D Group**	**Control Group**	***P***^**a**^
	**(n=22)**	**(n=21)**	
Age (years)	20.1±1.1^b^	20.3±0.8	0.50
Gender	21 F, 1 M	18 F, 2 M	0.52
Height (cm)	158.8±6.8	160.3±5.9	0.46
BMI (kg/m^2^)	26.0±1.8	25.7±1.7	0.66
Obese/overweight (%)^c^	22.2	10.5	0.67
Energy (kcal/d) ^a^	1907.3±206.3	1888.0±349.5	0.83
Protein (%)	14.1±2.6	14.7±2.4	0.41
Fat (%)	31.7±4.4	30.7±6.7	0.53
Carbohydrates (%)	54.2±5.4	54.6±7.0	0.82
Habitual Calcium Intake (mg/d)	426.5±152.2	392.1±141.1	0.45

Calcium+D, Calcium+Vitamin D_3_. BMI, body mass index. VFA, visceral fat area.

^a^ Independent t-test between means.

^b^ Mean ± standard deviation (all such values).

^c^ Overweight was defined as BMI from 24 to 27.9 kg/m^2^, and obesity was defined as BMI≥28 kg/m^2^[14-16].

**Table 2:** Anthropometric and metabolic variables before and after intervention in variables throughout the study

	**Calcium+D (n=22)**		**Control (n=21)**		***P***^**a**^		
	**Pre**	**Post**	**Pre**	**Post**	**Time**	**Treatment**	**Time×Treatment (adjusted**^**b**^**)**
Body weight (kg)	65.7±7.6 ^c^	61.5±7.2	66.3±8.3	62.8±9.7	<0.001	0.69	0.25 (0.16)
BMI (kg/m^2^)	26.0±1.8	24.2±1.8	25.7±1.7	24.2±2.2	<0.001	0.86	0.22 (0.19)
Fat mass (kg)	20.4±3.3	17.6±3.5	19.8±3.1	17.9±3.9	<0.001	0.73	0.02 (0.01)
Fat percentage (%)	31.0±3.0	28.4±3.5	29.9±2.8	28.4±3.0	<0.001	0.55	0.02 (0.02)
Lean mass (kg)	41.6±5.2	40.5±4.5	42.8±5.9	41.3±6.2	<0.001	0.54	0.31 (0.43)
Visceral fat mass (kg) ^d^	2.2±0.5	1.7±0.4	2.1±0.5	1.9±0.6	<0.001	0.75	0.01 (0.01)
VFA (cm^2^) ^d^	56.6±12.2	44.6±12.6	54.7±13.4	48.2±15.5	<0.001	0.85	0.02 (0.02)
Waist (cm)	81.5±4.1	75.2±5.3	80.9±5.6	76.3±6.8	<0.001	0.85	0.34 (0.26)
Systolic BP (mmHg) ^d^	119.2±10.5	109.6±9.9	123.0±10.5	111.9±10.4	<0.001	0.20	0.64 (0.67)
Diastolic BP (mmHg) ^d^	70.7±7.1	64.2±4.7	70.0±7.8	65.4±6.3	0.001	0.79	0.58 (0.62)
Glucose (mmol/L)	4.62±0.26	4.89±0.37	4.55±0.34	4.91±0.31	<0.001	0.96	0.22 (0.16)
Insulin (mIU/L)	10.49±5.04	9.24±5.36	9.07±4.02	9.23±4.62	0.58	0.52	0.44 (0.41)
TC (mmol/L)	3.89±0.52	3.78±0.61	4.11±0.43	3.93±0.48	0.03	0.58	0.57 (0.42)
TG (mmol/L)	0.76±0.48	0.78±0.25	0.76±0.32	0.86±0.67	0.36	0.75	0.61 (0.61)
HDL (mmol/L)	1.49±0.39	1.59±0.36	1.66±0.34	1.51±0.30	0.22	0.53	0.11 (0.19)
LDL (mmol/L)	1.89±0.50	2.41±0.43	1.98±0.50	2.10±0.51	0.23	0.43	0.62 (0.75)

Calcium+D, Calcium+vitamin D_3_. VFA, visceral fat area. Waist, waist circumference BP, blood pressure. TC, total cholesterol. TG, triglyceride. HDL, high-density lipoproteins. LDL, low-density lipoproteins. Change values=[(mean values wk 12)-(mean values wk 0)].

^a^ Repeated measures analysis of variance.

^b^ Adjusted for age, initial calcium intake and baseline body weight.

^c^ Mean ± standard deviation (all such values).

^d^ n=20 and 19 for Calcium+D and control groups, respectively.

**Table 3:** Body composition changes between baseline and wk 4, wk 8, and wk 12

	**Δ**_**1**_**(wk0 ~ wk 4)**			**Δ**_**2**_**(wk0 ~ wk 8)**			**Δ**_**3**_**(wk0 ~ wk 12)**		
	**Calcium+D**	**Control**	***P***^**a**^	**Calcium+D**	**Control**	***P***	**Calcium+D**	**Control**	***P***
	**(n=26)**	**(n=25)**		**(n=25)**	**(n=23)**		**(n=22)**	**(n=21)**	
Body weight (kg)	−2.2±1.4 ^b^	1.7±1.2	0.16	−3.3±1.6	3.3±1.4	0.94	−4.1±1.8	−3.5±1.9	0.25
Fat mass (kg)	−1.7±1.7	−1.0±1.1	0.13	−2.4±1.2	−1.7±0.8	0.03	−2.8±1.3	−1.8±1.3	0.02
Fat percentage (%)	−1.6±2.5	−0.8±1.3	0.19	−2.1±1.2	−1.1±0.9	<0.01	−2.6±1.6	−1.4±1.5	0.02
Lean mass (kg)	−0.4±1.0	−0.5±1.2	0.77	−0.8±0.9	−1.2±1.7	0.24	−1.1±1.1	−1.4±1.2	0.31
Visceral fat mass (kg) ^c^	−0.3±0.3	−0.1±0.2	0.09	−0.4±0.3	−0.2±0.2	<0.01	−0.5±0.2	−0.3±0.2	0.01
VFA (cm^2^) ^c^	−6.6±7.6	−2.9±6.6	0.12	−10.9±7.2	−4.6±4.9	<0.01	−12.0±6.4	−6.5±7.2	0.02

Calcium+D, Calcium+vitamin D_3_ group. Control, control group. VFA, visceral fat area.

Δ_1_=[(mean values wk 4)-(mean values wk 0)]. Δ_2_=[(mean values wk 8)-(mean values wk 0)]. Δ_3_=[(mean values wk 12)-(mean values wk 0)].

^a^ The *P*-value was calculated by independent t-test between means.

^b^ Mean ± standard deviation (all such values).

^c^ n=24 in both groups between wk 0 and wk4; n=23 and 22 for Calcium+D and control groups, respectively, between wk 0 and wk8; n=20 and 19 for Calcium+D and control groups, respectively, between wk 0 and wk12.

Energy and nutrient intake at baseline were not significantly different between groups. At the end of the study, the estimated daily intake of energy was significantly lower in both groups (−393.9±247.8 kcal in the calcium + D group and −307.9±298.2 kcal in the control group). Changes in percentage of energy from protein, fat and carbohydrates were 0.9±2.2%, - 1.9±3.9%, and 1.0±5.2%, respectively, in the calcium + D group and −0.1±2.3%, - 0.3±4.7%, and 4.4±5.5%, respectively, in the control group. No significant difference was found in all these dietary changes between groups (*P*>0.05 for all). Dietary calcium intake and composition of macronutrients did not change significantly in both groups.

## Discussion

In the present study, we observed that calcium+D supplementation for 12 weeks facilitated fat loss in very-low calcium consumers independent of energy restriction, which is concordant with other randomized controlled trials [14,16-20], suggesting a beneficial effect of calcium intake on weight management.

To our knowledge, ours is among the few relevant studies to evaluate the effect of combined calcium and vitamin D_3_ administration in very-low calcium consumers by setting initial calcium intake at <600 mg/day as one of the inclusion criteria. The mean initial calcium intake of subjects was estimated to be 409.7 mg/day, below the threshold previously suggested [10] to predict promising outcomes of a weight-loss program. Previously published studies were carried out mostly in Western countries, where recruitment of participants with very-low calcium consumption was difficult because of the moderate level of calcium intake among the majority of Western populations: 856 mg/day for men and 670 mg/day for women in American adults; moreover, half of them use calcium supplements [21]. Whereas in the Chinese population, the average intake of calcium was around 390 mg/day, according to the Chinese National Nutrition and Health survey in 2002 [22]. Therefore, this population provides a stable sample of very-low calcium consumers for investigating the potential anti-adiposity effect of calcium below the cut-off value of 600 mg/day. As expected, we observed significant augmentation of body fat loss and visceral fat loss in the calcium and vitamin D_3_ supplemented group after the intervention. In contrast, a 24-month trial [23] found no significant difference in anthropometric variables between the calcium-supplemented group and placebo in subjects with initial calcium intake of 882 mg/d. Neither did Manios et al. [24] or Wagner et al. [25] observed any beneficial effect of calcium (with or without vitamin D) on weight management in subjects with over 650 mg/day of initial calcium intake, which constitutes one explanation for the inconsistency. The dosage used in calcium-supplemented programs was diverse. Zemel and his group [17] detected a beneficial effect of calcium carbonate, an additional 800 mg/day, on weight reduction, which was increased by 26% compared with the control subjects. In Faghih’s study [19], supplementation of 800 mg/day was also found to be effective in facilitating weight loss. Major et al. [16] reported a significantly greater decrease in body weight and fat mass in the calcium+D group (5.8 vs. 1.4 kg and 4.7 vs. 1.2 kg, respectively), receiving 1200 mg calcium carbonate and 400 IU vitamin D daily. In the present study, however, the dose was administered as one tablet (600 mg calcium carbonate, 125 IU vitamin D) daily. We used a relatively lower dose because the initial calcium intake in the majority of Chinese is low. In most relevant studies carried out in Western populations the average calcium intake was around 600 to 800 mg/d (even higher in some studies), and the dose was about 1.5 times the initial calcium intake. And therefore, in the current study, the additional 600 mg/d was administered in subjects with initial calcium intake of around 400 mg/d. Besides, 125 IU of vitamin D_3_, which is linked with 600 mg of calcium in each tablet, was administered in the present study, since it is an over-the-counter supplement.

The current study recruited obese and overweight subjects with a lower body mass index (BMI) cutoff, which was believed to be required for Chinese population [26,27], who has a lower baseline BMI to begin with and the risk of cardiovascular diseases is doubled at BMI of 23.0 to 24.9, and tripled at BMI of 25.0 to 26.9 [28]. In addition, we recruited subjects aged 18 to 25 years from colleges, because these students usually had similar dietary patterns and their compliance with dietary instructions was fairly good. This made it possible for us to evaluate the potential difference between an energy-restricted diet with calcium+D supplementation and energy restriction alone in a short period of 12 weeks. Actually, the compliance to the weight loss program was very good, since at the end of the study, subjects in both groups achieved 5~6% of body weight reduction, suggesting they were motivated by the intervention. In fact, reported energy intake was significantly lower in both groups at endpoint versus baseline. It is worth mentioning that the calcium+D group achieved 55.6% augmentation of fat mass loss compared with the control, despite the fact that there was no significant difference in body weight change between groups. That means the calcium+D group lost more adipose tissue during energy restriction.

The greater decrease in fat mass observed in the calcium+D group of the current study could result from several factors attributing to calcium metabolism. First, a calcium-rich diet is shown to increase fat oxidation [29], promote fat cell apoptosis [10], and reduce lipid absorption due to the formation of insoluble calcium-fatty acid soaps in the intestine, which are eventually excreted in the feces [30]. Second, high dietary calcium intake is associated with suppression of 1,25-dihydroxyvitamin D (1,25-(OH)_2_D) levels which in turn act to decrease calcium influx into the cell. These modifications consequently stimulate lipolysis and inhibit lipogenesis in the adipocyte [10,14]. Third, a calcium-specific appetite control, proposed by Tordoff, could have played a role in facilitating fat loss in a calcium-supplemented program [16].

Moreover, the findings of the current study are concordant with the majority of previously published clinical trials showing no significant differences between treatment and control subjects in glucose and insulin profiles [31] and in circulating lipids and lipoprotein concentrations [32-34]. There is only one previous study [35] reporting a significantly greater decreases in total:LDL, LDL:HDL (*P*<0.01 for both) and LDL cholesterol (*P*<0.05) after 15 weeks of intervention in the active group, receiving a supplementation of 1200 mg/day calcium and 400 IU vitamin D and a caloric deficit of 700 kcal/day, than in the placebo group, receiving 700 kcal/day of energy restriction alone. In addition, the present study showed no significant impact of additional elemental calcium+D on blood pressures. It seems that the antihypertensive effect of calcium may be more pronounced with dairy-derived calcium [36,37] than elemental calcium [32,34]. The former was examined in studies that carried out over short terms (5 weeks to 16 weeks), while the latter was used in moderate or long-term studies (6 months and 2 years). If that is the case, it is not surprising that we did not find any significant changes in blood pressure in such a short term. Besides, other minerals (potassium and magnesium) and bioactive peptides in dairy, which act as angiotensin converting enzyme inhibitors [38], may also explain the antihypertensive effect in intervention trials. It will be interesting to conduct further research on the whey-derived bioactive peptides for blood pressure management.

The primary limitation of our study is that participants in the control group were not given a placebo due to budget constraints. However, in the present study the dietitians, supervising and giving dietary instructions to subjects, were not involved in any other procedures such as randomization allocation or evaluation of outcome measurements. Subjects in the present study made no communications with each other for their participation is confidential and individual. In addition, we avoided reporting any subjective measurements, such as hunger scores, as well, so as to minimize bias that could occur in an open-label trial. Not using dual energy X-ray absorptiometry (DEXA), the gold standard method for assessing body composition, constitutes another limitation. However, BIA is also a validated and reliable method to assess body composition [13,39]. Other limitations include the primarily female composition of the study sample, the relatively small dose of vitamin D_3_ used. From a statistical perspective, the beneficial effects of calcium+D supplementation on fat loss could be attribute to calcium as much as to vitamin D_3_. However, previously published literature tends to attribute a large contribution to calcium instead of vitamin D due to the controversial results and lack of evidence of the effect of vitamin D on adiposity [35]. Future research in this area should be oriented toward a better understanding of the dose–response effect of calcium supplementation (with or without vitamin D) on weight management by administering different dosages of this mineral.

## Conclusions

To summarize, our results showed supplementation of calcium plus vitamin D_3_ for 12 weeks facilitated body fat and visceral fat loss during energy restriction in overweight or obese very-low calcium consumers.

## Abbreviations

calcium+D: Calcium plus vitamin D_3_; wk: Week; AgRP/NPY: Agouti Related Protein/ Neuropeptide Y; POMC/CART: Pro-Opiomelanocortin/ Cocaine- Amphetamine- Regulated Transcription; 1,25-(OH)_2_D: 1,25-dihydroxyvitamin D; BMI: Body mass index; FFQ: Food frequency questionnaire; TDEE: Total daily energy expenditure; BMR: Basal metabolic rate; BIA: Bioelectric impedance analysis; VFA: Visceral fat area; TC: Total cholesterol; TG: Triglyceride; HDL: High-density lipoproteins; LDL: Low-density lipoproteins; CDC: The Chinese Center for Disease Control and Prevention; DEXA: Dual energy X-ray absorptiometry; ITT: Intention-to-treat.

## Competing interests

None of the authors have any conflict of interest to declare.

## Authors’ contributions

WZ designed and conducted the study, and drafted the manuscript. YW carried out the randomization procedure. QH, YQ, and SM participated in the project as coordinators, giving dietary instructions to subjects and participating in data collection. DC provided technical support throughout the program and had primary responsibility for final content. NL and SA participated in revising the manuscript. All authors read and approved the final manuscript.
