# Correction: Calcium plus vitamin D3 supplementation facilitated Fat loss in overweight and obese college students with very-low calcium consumption: a randomized controlled trial

**DOI:** 10.1186/1475-2891-12-43

**Published:** 2013-04-08

**Authors:** Wei Zhu, Donglian Cai, Ying Wang, Ning Lin, Qingqing Hu, Yang Qi, Shuangshuang Ma, Sidath Amarasekara

**Affiliations:** 1 Department of Nutrition, Shanghai Institute of Health Sciences, Shanghai, 201318, China; 2 Department of Clinical Nutrition, Changhai Hospital, Second Military Medical University, Shanghai, 200433, China; 3 Department of Clinical Nutrition, Chengdu Military General Hospital, Chengdu, 610083, China

## Correction

After publication of this article [1], we noted errors to the “body weight” row of Table three. The values for the controls of Δ_1_(wk 0 ~ wk 4) and Δ_2_(wk 0 ~ wk 8) should be negative (please see Table 1 below, a corrected version of Table three).

**Table 1:** Body composition changes between baseline and wk 4, wk 8, and wk 12

	**Δ**_**1**_**(wk0 ~ wk 4)**			**Δ**_**2 **_**(wk0 ~ wk 8)**			**Δ**_**3 **_**(wk0 ~ wk 12)**		
	**Calcium+D (n = 26)**	**Control (n = 25)**	***P***^**a**^	**Calcium+D (n = 25)**	**Control (n = 23)**	***P***	**Calcium+D (n = 22)**	**Control (n = 21)**	***P***
Body weight (kg)	−2.2 ± 1.4 ^b^	−1.7 ± 1.2	0.16	−3.3 ± 1.6	−3.3 ± 1.4	0.94	−4.1 ± 1.8	−3.5 ± 1.9	0.25
Fat mass (kg)	−1.7 ± 1.7	−1.0 ± 1.1	0.13	−2.4 ± 1.2	−1.7 ± 0.8	0.03	−2.8 ± 1.3	−1.8 ± 1.3	0.02
Fat percentage (%)	−1.6 ± 2.5	−0.8 ± 1.3	0.19	−2.1 ± 1.2	−1.1 ± 0.9	<0.01	−2.6 ± 1.6	−1.4 ± 1.5	0.02
Lean mass (kg)	−0.4 ± 1.0	−0.5 ± 1.2	0.77	−0.8 ± 0.9	−1.2 ± 1.7	0.24	−1.1 ± 1.1	−1.4 ± 1.2	0.31
Visceral fat mass (kg) ^c^	−0.3 ± 0.3	−0.1 ± 0.2	0.09	−0.4 ± 0.3	−0.2 ± 0.2	<0.01	−0.5 ± 0.2	−0.3 ± 0.2	0.01
VFA (cm^2^) ^c^	−6.6 ± 7.6	−2.9 ± 6.6	0.12	−10.9 ± 7.2	−4.6 ± 4.9	<0.01	−12.0 ± 6.4	−6.5 ± 7.2	0.02

Calcium+D, Calcium+vitamin D_3_ group. Control, control group. VFA, visceral fat area.

Δ_1_=[(mean values wk 4)-(mean values wk 0)]. Δ_2_=[(mean values wk 8)-(mean values wk 0)]. Δ_3_=[(mean values wk 12)-(mean values wk 0)].
